# Integrative Analysis From Multicenter Studies Identifies a WGCNA-Derived Cancer-Associated Fibroblast Signature for Ovarian Cancer

**DOI:** 10.3389/fimmu.2022.951582

**Published:** 2022-07-08

**Authors:** Songwei Feng, Yi Xu, Zhu Dai, Han Yin, Ke Zhang, Yang Shen

**Affiliations:** ^1^ Department of Obstetrics and Gynaecology, Zhongda Hospital, School of Medicine, Southeast University, Nanjing, China; ^2^ State Key Laboratory of Bioelectronics, School of Biological Science and Medical Engineering, Southeast University, Nanjing, China

**Keywords:** cancer-associated fibroblasts, WGCNA, ovarian cancer, prognosis, tumor microenvironment

## Abstract

Cancer-associated fibroblasts (CAFs) are a major contributor to tumor stromal crosstalk in the tumor microenvironment (TME) and boost tumor progression by promoting angiogenesis and lymphangiogenesis. This study aimed to identify prognostic genes associated with CAFs that lead to high morbidity and mortality in ovarian cancer (OC) patients. We performed bioinformatics analysis in 16 multicenter studies (2,742 patients) and identified CAF-associated hub genes using the weighted gene co-expression network analysis (WGCNA). A machine learning methodology was used to identify COL16A1, COL5A2, GREM1, LUM, SRPX, and TIMP3 and construct a prognostic signature. Subsequently, a series of bioinformatics algorithms indicated risk stratification based on the above signature, suggesting that high-risk patients have a worse prognosis, weaker immune response, and lower tumor mutational burden (TMB) status but may be more sensitive to routine chemotherapeutic agents. Finally, we characterized prognostic markers using cell lines, immunohistochemistry, and single-cell sequencing. In conclusion, these results suggest that the CAF-related signature may be a novel pretreatment guide for anti-CAFs, and prognostic markers in CAFs may be potential therapeutic targets to inhibit OC progression.

## Introduction

Cancer-associated fibroblasts (CAFs) play a key role in the tumor microenvironment (TME) and influence tumor progression and metastasis through multiple pathways, including remodeling of the extracellular matrix (ECM), producing growth factors, and promoting angiogenesis (1). Meanwhile, ovarian cancer (OC) is a heterogeneous disease characterized by a propensity for peritoneal spread. Due to the complex interconnected signaling network and the unique peritoneal TME, cancer cells can interact with CAFs, adipocytes, immune cells, and chemokines (2). As a result, tumor migration and immune evasion frequently occur in OC patients, and immunotherapy has little effect (3).

The ECM is composed and reconstituted by CAFs, a barrier that supports tumor cell invasion and inhibits infiltration of antitumor immune cells, thus leading to immune evasion and chemoresistance (4, 5). Several researchers have explored different CAF subgroups with varying CAF marker expressions, such as alpha-smooth muscle actin (α-SMA), fibronectin attachment protein (FAP), and pelleted growth factor receptor (PDGFR) (6, 7). For example, in oral cancer, WNT2^+^ CAFs were negatively correlated with CD8^+^ T-cell activity (8). In pancreatic cancer, knocking down α-SMA^+^ CAFs unexpectedly enhanced tumor infiltration and increased Regulatory T cells (Tregs) abundance, leading to enhanced disease progression and reduced survival rates in mice (9). In breast and colon cancer, DNA-based vaccines targeting FAP induced the killing of CAFs by CD8^+^ T cells (10). Therefore, targeting the CAF-mediated immunosuppressive stromal microenvironment in combination with immunotherapy is expected to improve immune checkpoint inhibitor (ICI) response (11).

Weighted gene co-expression network analysis (WGCNA) is a systematic bioinformatics algorithm that enables the integration of highly correlated genes into several modules (12). This is a novel method to explore the relationship between numerous genes and clinical phenotypes. WGCNA has been applied to identify CAF markers, such as in gastric cancer (13), bladder cancer (14), and renal cell carcinoma (15). However, to date, CAFs have not been analyzed by WGCNA in large-sample multicenter OC cohorts. In this study, we integrated 16 multicenter studies that included 2,742 patients with complete follow-up information for bioinformatics analysis. We explored the hub modules most relevant for CAF infiltration and identified COL16A1, COL5A2, GREM1, LUM, SRPX, and TIMP3 as prognostic CAF markers. Subsequently, CAF signatures capable of predicting prognosis and treatment response were constructed, and the predictive ability was validated in multiple cohorts. In addition, we characterized markers using cell lines, immunohistochemistry, and single-cell sequencing. In conclusion, our results imply that the CAF signature may be a novel anti-CAF therapeutic approach in OC.

## Methods

### Datasets and Data Preprocessing

The fragments per kilobase of transcript per million mapped reads (FPKM) format RNA sequencing (RNA-seq) data with complete follow-up information of 372 samples were downloaded from The Cancer Genome Atlas (TCGA) database (16). Except for the samples without survival follow-up information, we still retained the samples with other clinical information missing. The somatic mutation data were also acquired from TCGA database. The tumor mutational burden (TMB) value of each sample was calculated *via* the tmb algorithm in the “maftools” package (17). We performed log2 [transcripts per million (TPM) + 1] transformation on the above raw data (18). In the Gene Expression Omnibus (GEO) database (19), we integrated the multiple datasets (RNA-seq or microarray) based on the GPL570 platform (GSE19829, GSE18520, GSE9891, GSE26193, GSE30161, and GSE63885; n = 597), GPL96 platform (GSE3149, GSE23554, GSE26712, and GSE14764; n = 409), GPL7759 platform (GSE13876, n = 415), GPL2986 platform (GSE49997, n = 194), and GPL14951 platform (GSE140082, n = 380). In addition, we downloaded anti-programmed death-1 (PD-1) dataset (IMvigor, n = 348) and anti-PD-L1 dataset (GSE78220, n = 27) based on immunotherapy. Cell line RNA-seq data from 47 fibroblast origins and 37 OC origins were acquired from the Cancer Cell Line Encyclopedia (CCLE) database (20). Immunohistochemical (IHC) staining images in OC tissues were downloaded from the Human Protein Atlas (HPA) database (21). Batch effects from meta-cohorts (GPL570 or GPL96) were corrected using the ComBat algorithm in the “sva” package (22). CAF markers were collected from previous references (23).

In conclusion, we integrated 16 multicenter studies and included 2,742 patients with complete follow-up information for our bioinformatics analysis.

### Cancer-Associated Fibroblasts and Stromal Analysis

CAF abundances and stromal scores were computed using four methods: Estimate the Proportion of Immune and Cancer cells (EPIC) algorithm (24), xCell algorithm (25), microenvironment cell populations-counter (MCP-counter) algorithm (26), and Estimation of Stromal and Immune cells in Malignant Tumor tissues using Expression data (ESTIMATE) algorithm (27). We used “IOBR” package to invoke the above algorithm (28). The CAF abundances calculated by EPIC and MCP-counter were defined as phenotypic data for subsequent WGCNA. The data calculated by other algorithms were used for validation.

### Weighted Gene Co-Expression Network Analysis

The “WGCNA” package (12) screened hub genes that were significantly associated with CAF scores. The expression profiles of the top 25% of the variance in the GPL570 meta-cohort and TCGA-OV cohort first were as the input. Then, according to our previous study (29), a soft threshold was determined, an adjacency matrix was clustered, and a hub module was determined. The strongest positive correlation was selected for further analysis by calculating the Pearson correlation coefficient between the modules and CAF scores. Then, we measured gene significance (GS) for each gene’s traits and module membership (MM) in the hub module. Finally, genes in the module were screened as potential CAF-related genes using MM >0.6 and GS >0.6 as thresholds.

### Enrichment Analysis

The h.all.v7.4.symbols gene set was downloaded from the MSigDB database (30) for enrichment analysis in “GSVA” package (31). The adj.P value <0.05 was considered statistically significant. Gene Ontology (GO) and Kyoto Encyclopedia of Genes and Genomes (KEGG) analyses were conducted using “clusterProfiler” package (32). The adj.P-value <0.05 and adj.q-value <0.05 were considered statistically significant.

### Construction and Validation of the Cancer-Associated Fibroblast Signature

The GPL570 meta-cohort with a larger sample size was used as the training cohort, and other cohorts were used as the validation cohort. Univariate Cox regression analysis was performed on common hub genes in 16 multicenter studies (P-value <0.05). In the least absolute shrinkage and selection operator (LASSO) regression analysis (33), 1,000 iterations were performed to reduce the genes, and subsequently, the above genes were subjected to multivariate Cox regression analysis to obtain the coefficients. CAF risk score was derived using the same formula as in our previous study (34, 35). The OC patients in each cohort were divided into high-risk and low-risk groups, and the cutoff value for each cohort was used as the threshold.

### Chemotherapy Response Predictions

The “pRRophetic” package (36) was used to calculate IC50 value (bleomycin, cisplatin, docetaxel, gemcitabine, doxorubicin, and etoposide) of different samples based on gene expression.

### Single-Cell Sequencing Analysis

We analyzed single-cell RNA-sequencing (scRNA-seq) data (GSE118828) from OC tissues based on the Tumor Immune Single Cell Hub (TISCH) database (37), and the whole cells were annotated into six clusters: fibroblasts, myofibroblasts, endothelial, malignant, monocyte or macrophage (Mono/Macro), and conventional CD4 T cell (CD4Tconv).

### Immunofluorescence Staining

In total, two formalin-fixed paraffin-embedded (FFPE) tissue (primary tumors and recurrent tumors) blocks were selected from the Zhongda Hospital Southeast University. Immunofluorescence staining was also done by a commercial entity (Servicebio, Wuhan, Hubei, China). According to the company, detailed methods are available in a previous publication (38). Antibody staining order always remains the same, all slices with 4,6-diamidino-2-phenylindole (DAPI) (Servicebio, G1012) finally after dyeing. Monoclonal antibodies in immunofluorescence panels were CD8 (Servicebio, GB13068-2, 1:500), Foxp3 (Servicebio, GB112325, 1:3,000), and α-SMA (Servicebio, GB13044, 1:1,000). Slices were placed under a scanner to collect images, and image data were obtained using CaseViewer software.

### Statistical Analysis

All statistical analyses were performed using the R software (v.4.0.1). The Wilcoxon test was applied for pairwise comparisons. The Kaplan–Meier analysis with the log-rank test was adopted for overall survival comparisons. More detailed statistical methods for transcriptome data processing are covered in the above section. P < 0.05 was considered statistically significant.

## Results

### Cancer-Associated Fibroblasts and Stromal Score Could Be Considered Prognostic Markers for Ovarian Cancer

**Figure 1:**
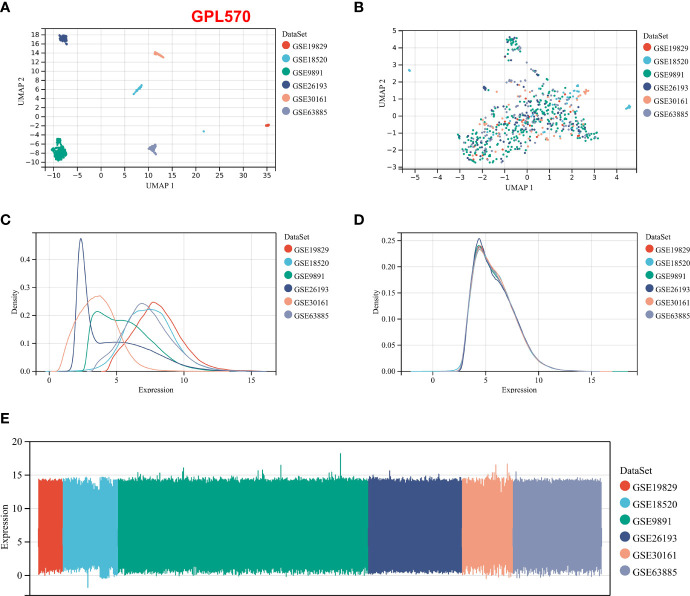
Normalization process based on the GPL570 platform dataset. **(A)** UMAP plot of the six datasets before normalization. **(B)** UMAP plot of the six datasets after normalization. **(C)** Expression density plot of the six datasets before normalization. **(D)** Expression density plot of the six datasets after normalization. **(E)** Expression distribution plots for the six datasets after normalization. UMAP, Uniform Manifold Approximation and Projection.

**Figure 2:**
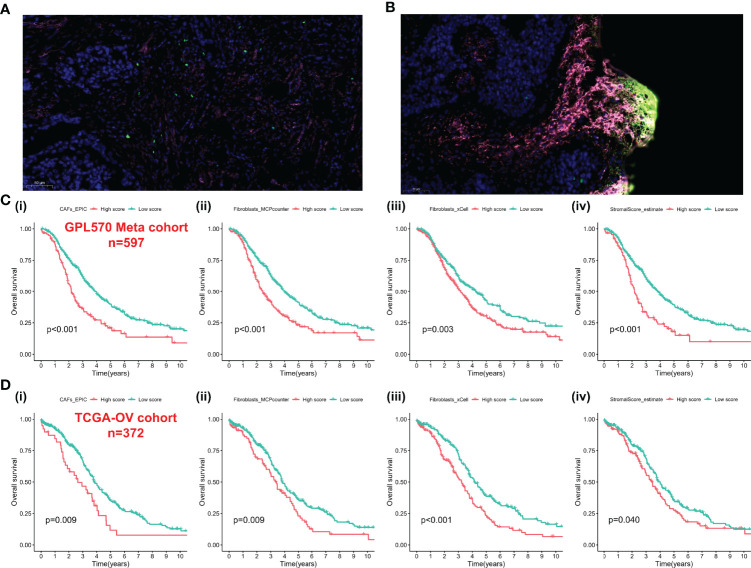
CAFs spatially associate with Treg cells and survival analysis-based CAFs and stromal score. **(A)** Immunofluorescence staining of the original ovarian tissue samples. **(B)** Immunofluorescence staining results of recurrent ovarian tissue samples. **(C)** The Kaplan–Meier analysis of GPL570 meta-cohort, including CAF_EPIC (i), Fibroblasts_MCPcounter (ii), Fibroblasts_xCell (iii), and StromalScore_estimate (iv). **(D)** The Kaplan–Meier analysis of TCGA-OV cohort, including CAF_EPIC **(i)**, Fibroblasts_MCPcounter (ii), Fibroblasts_xCell (iii), and StromalScore_estimate (iv). CAFs, Cancer-Associated Fibroblasts; Tregs, Regulatory T cells.

We integrated the multidatasets based on the GPL570 platform (GSE19829, GSE18520, GSE9891, GSE26193, GSE30161, and GSE63885), and the Uniform Manifold Approximation and Projection (UMAP) analysis showed the distribution of each dataset before and after removal of batch effect (**Figures 1A, B**). The expression density plot also revealed that the batch effect of the GPL570 meta-cohort was well removed (**Figures 1C, D**). Finally, we normalized the expression profiles of 597 samples with complete follow-up information (**Figure 1E**). Previous references have reported the ability of CAFs to recruit immunosuppressive cells, so we performed immunofluorescence staining using immunofluorescence in patients with primary tumors and in patients with recurrent tumors (39). Interestingly, there was a recruitment of Treg cells (green) around the CAF cells (pink) in patients with primary tumors (**Figure 2A**), especially in the recurrent samples, where a larger number of Treg cells clustered to the prominent part of the CAF cells (**Figure 2B**). CD8^+^ cells (red) were rarely seen around CAF cells in both samples. Subsequently, the CAF infiltration score was predicted by EPIC, xCell, and MCP-counter algorithms based on the GPL570 meta-cohort (n = 597) and TCGA-OV cohort (n = 372), and the stromal score was calculated by ESTIMATE algorithm. We divided all samples into a high CAF/stromal score group and a low CAF/stromal group according to the cutoff values of the scores calculated by the four bioinformatics algorithms. In the GPL570 meta-cohort, the results showed that higher CAF infiltration and stromal score were significantly associated with poorer overall survival (OS) in OC patients (**Figure 2C**). Similarly, it could also be used as a predictive biomarker in TCGA-OV cohort (**Figure 2D**). Our study defined the CAF abundances calculated by EPIC and MCP-counter as phenotypic data for subsequent WGCNA. The data calculated by other algorithms were used for validation.

### Co-Expression Network of Cancer-Associated Fibroblast Scores

**Figure 3:**
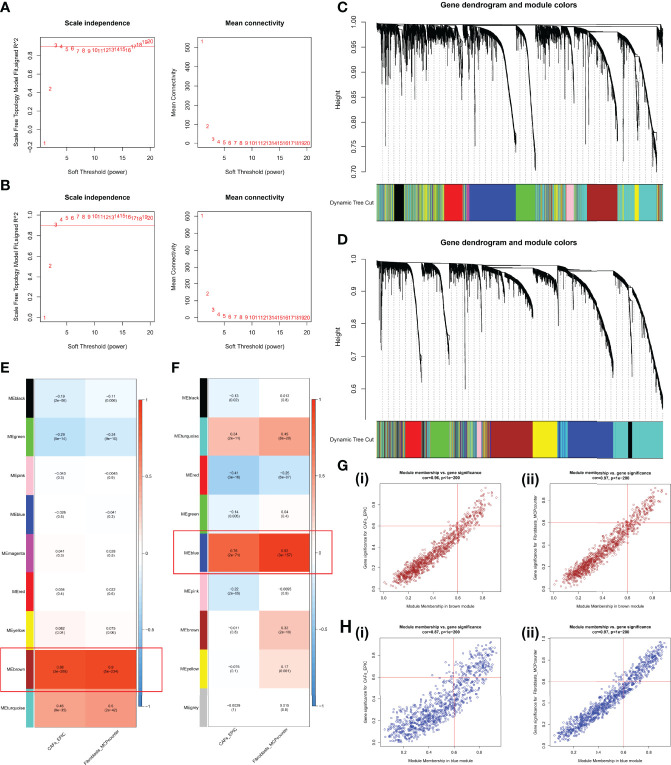
WGCNA in the GPL570 meta-cohort and TCGA-OV cohort. **(A)** Scale independence and mean connectivity in the GPL570 meta-cohort. **(B)** Scale independence and mean connectivity in TCGA-OV cohort. **(C)** Gene dendrogram and modules before merging in the GPL570 meta-cohort. **(D)** Gene dendrogram and modules before merging in TCGA-OV cohort. **(E)** Pearson correlation analysis of merged modules and CAF score in the GPL570 meta-cohort. **(F)** Pearson correlation analysis of merged modules and CAF score in TCGA-OV cohort. **(G)** Scatterplot of MM and GS from the brown module in the GPL570 meta-cohort, including CAFs_EPIC (i) and Fibroblasts_MCPcounter (ii). **(H)** Scatterplot of MM and GS from the blue module in TCGA-OV cohort, including CAFs_EPIC (i) and Fibroblasts_MCPcounter (ii). WGCNA, Weighted Gene Co-expression Network Analysis; CAFs, Cancer-Associated Fibroblasts; GS, Gene Significance; MM, Module Membership.

WGCNA was performed using the expression profiles of the top 25% of variance in the GPL570 meta-cohort and TCGA-OV cohort. The soft threshold power in the GPL570 meta-cohort was 3 (**Figure 3A**); similarly, the threshold for TCGA-OV cohort was also 3 (**Figure 3B**). Subsequently, dynamic module identification was performed in the different cohorts, with the number of genes per module not less than 50 (**Figures 3C, D**). For the GPL570 meta-cohort, 9 co-expression modules were clustered, with the brown module having the strongest positive correlation with CAFs_EPIC score (Cor = 0.88, P = 3e-208) and Fibroblasts_MCPcounter score (Cor = 0.9, P = 5e-234) (**Figure 3E**). For TCGA-OV cohort, the 9 co-expression modules were clustered, with the blue module having the strongest positive correlation with CAFs_EPIC score (Cor = 0.76, P = 2e-71) and Fibroblasts_MCPcounter score (Cor = 0.92, P = 3e-157) (**Figure 3F**). In the brown module, positive correlations between CAFs_EPIC score (Cor = 0.96) and Fibroblasts_MCPcounter score (Cor = 0.97) were observed between MM and GS (**Figure 3G**); in the black module, positive correlations between CAFs_EPIC score (Cor = 0.87) and Fibroblasts_MCPcounter score (Cor = 0.97) were also observed between MM and GS (**Figure 3H**). Finally, 120 genes in the brown module and 160 genes in the blue module were screened as potential CAF-related genes using MM > 0.6 and GS > 0.6 as thresholds.

### Functional Analyses of Cancer-Associated Fibroblast-Related Genes

**Figure 4:**
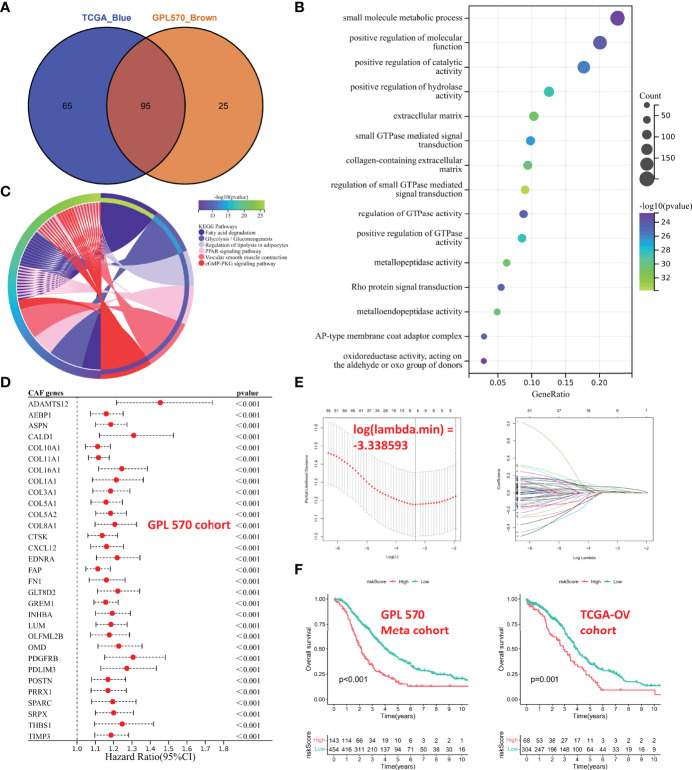
Functional analyses and construction of the CAF-based signature. **(A)** The hub CAF-related genes were overlapped in the brown module and the blue module. **(B)** GO enrichment analysis. **(C)** KEGG enrichment analysis. **(D)** Univariate Cox regression analysis of common hub genes (P < 0.001). **(E)** LASSO regression analysis. **(F)** Kaplan–Meier analysis of different cohorts. On the left is GPL570 meta-cohort; on the right is TCGA-OV cohort. CAFs, Cancer-Associated Fibroblasts; GO, Gene Ontology; KEGG, Kyoto Encyclopedia of Genes and Genomes; LASSO, Least Absolute Shrinkage and Selection Operator.

The above CAF-related genes were overlapped and screened to 95 hub genes (**Figure 4A**). Regulation of small GTPase-mediated signal transduction, extracellular matrix, collagen-containing extracellular matrix, and metallopeptidase activity were the main enriched GO terms (**Figure 4B**). Fatty acid degradation, glycolysis/gluconeogenesis, regulation of lipolysis in adipocytes, peroxisome proliferator activated receptor (PPAR) signaling pathway, vascular smooth muscle contraction, and cyclic guanosine monophosphate (cGMP)/protein kinase G (PKG) signaling pathway were the mainly enriched KEGG pathways (**Figure 4C**).

### Construction of the Cancer-Associated Fibroblast-Based Signature

The GPL570 meta-cohort with a larger sample size was used as the training cohort, and TCGA-OV cohort was used as the validation group. Univariate Cox regression analysis was performed on common hub genes in the training cohort (**Figure S1**), with OS and survival time as dependent variables, and 63 prognostic genes (P < 0.05) were screened, and only some with P < 0.001 were shown in **Figure 4D**. The 63 prognostic genes were subjected to LASSO regression analysis to determine the minimum λ value (**Figure 4E**). Finally, 6 genes were identified for the CAF-based signature: CAF risk score = COL16A1 expression * 0.0924 + COL5A2 expression * -0.0031 + GREM1 expression * 0.0847 + LUM expression * 0.0069 + SRPX expression * 0.0649 + TIMP3 expression * 0.0425. The OC patients in each cohort were divided into high-risk and low-risk groups, and the cutoff for each cohort was used as the threshold value (GPL570 meta-cohort = 1.257016302; TCGA-OV cohort = 0.415034301). Kaplan–Meier curves showed that patients in the high-risk group had worse OS than that of those in the low-risk group (**Figure 4F**). These results suggested that the CAF signature was the hub prognostic marker for OC patients.

### Cancer-Associated Fibroblast-Based Signature Genes Were Correlated With Cancer-Associated Fibroblast Markers

**Figure 5:**
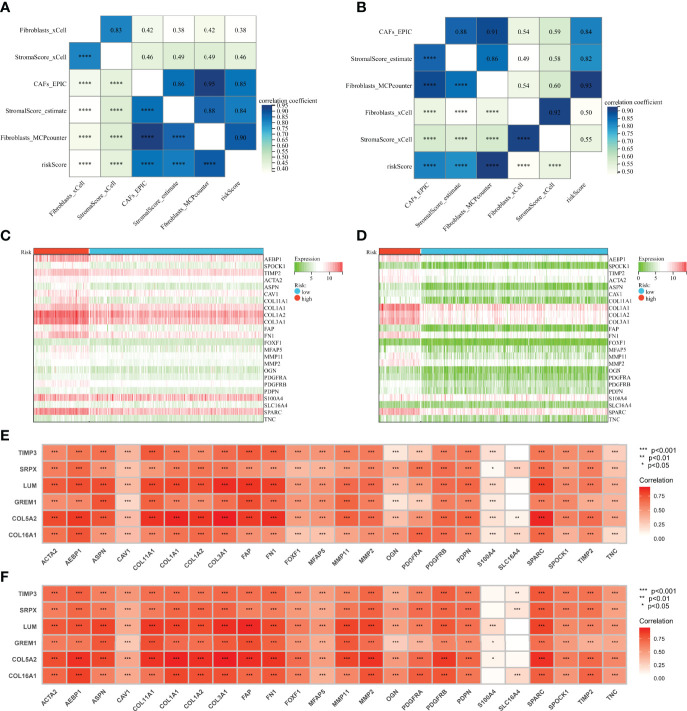
Genes involved in the signature were correlated with CAF markers. **(A)** Correlation analysis of CAF score, stromal score, and risk score in the GPL570 meta-cohort. **(B)** Correlation analysis of CAF score, stromal score, and risk score in TCGA-OV cohort. **(C)** Heatmaps of expression of CAF markers in different risk groups (GPL570 meta-cohort). **(D)** Heatmaps of expression of CAF markers in different risk groups (TCGA-OV cohort). **(E)** Correlation analysis of genes involved in signature and CAF markers (GPL570 meta-cohort). **(F)** Correlation analysis of genes involved in signature and CAF markers (TCGA-OV cohort). CAFs, Cancer-Associated Fibroblasts.

Spearman correlation analyses were performed between the CAF risk score and the CAF score predicted by the other methods (xCell, EPIC, ESTIMATE, and MCP-counter). Subsequently, we observed a strong and positive correlation between risk scores and CAF infiltration and stromal score in both GPL570 meta-cohort (**Figure 5A**) and TCGA-OV cohort (**Figure 5B**). Moreover, CAF marker genes from previous references had a higher expression in the high-risk group (**Figures 5C, D**). In addition, the expression levels of 6 genes in the signature also were highly and positively correlated with CAF marker expression (**Figures 5E, F**).

### Multidimensional Validation in Multicenter Studies

**Figure 6:**
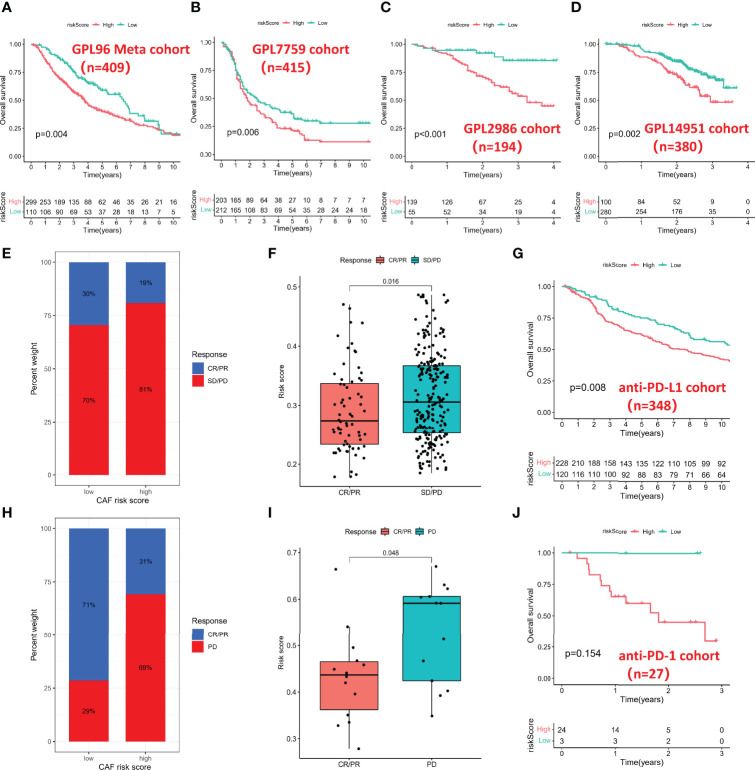
Multidimensional validation for risk score. **(A)** Kaplan–Meier analysis in the GPL96 meta-cohort. **(B)** Kaplan–Meier analysis in the GPL7759 cohort. **(C)** Kaplan–Meier analysis in the GPL2986 cohort. **(D)** Kaplan–Meier analysis in the GPL14951 cohort. **(E)** Histogram of anti-PD-L1 response distribution in different risk groups. **(F)** Box plot of risk score in different anti-PD-L1 response groups. **(G)** Kaplan–Meier analysis in the anti-PD-L1 cohort. **(H)** Histogram of anti-PD-1 response distribution in different risk groups. **(I)** Box plot of risk score in different anti-PD-1 response groups. **(J)** Kaplan–Meier analysis in the anti-PD-1 cohort. PD-1, Programmed Death-1; PD-L1, Programmed Cell Death 1 Ligand.

To further validate the prognostic value of the CAF-based signature, we integrated the GPL96 meta-cohort (GSE3149, GSE23554, GSE26712, and GSE14764) according to the method described above, which included a total of 409 patients (**Figure S2**). Meanwhile, the datasets based on GPL7759 (GSE13876, n = 415), GPL2986 (GSE49997, n = 194), and GPL14951 (GSE140082, n = 380) platforms were downloaded for external validation. The risk scores of each cohort were calculated with the same formula and stratified by their respective cutoff values (GPL96 meta-cohort = 0.976888643; GPL7759 cohort = 3.088372669; GPL2986 cohort = 0.147731773; GPL14951 cohort = 2.479072527). Unsurprisingly, risk score stratified patients by survival risk in multicenter studies, and OS was shorter in the high-risk group, such as in the GPL96 meta-cohort (**Figure 6A**, P = 0.004), GPL7759 cohort (**Figure 6B**, P = 0.006), GPL2986 cohort (**Figure 6C**, P < 0.001), and GPL14951 cohort (**Figure 6C**, P = 0.002).

### Cancer-Associated Fibroblast-Based Signature in the Role of Immunotherapy

Immunotherapy represented by PD-L1 and PD-1 blockade has undoubtedly become a breakthrough in cancer treatment, so we investigated whether the CAF-based signature could predict response to anti-PD-1 and anti-PD-L1 based on two immunotherapy cohorts. In the anti-PD-L1 cohort (IMvigor210), the high-risk group had a higher percentage of stable disease (SD)/progressive disease (PD). In contrast, more patients in the low-risk group were in complete response (CR)/partial response (PR) (**Figure 6E**). Moreover, patients with a low risk score exhibited a markedly prolonged survival (**Figure 6F**). In the anti-PD-1 cohort (GSE78220), the significant therapeutic advantages and clinical response in patients with a low score also were confirmed (**Figures 6H, I**). However, due to the small sample size of the anti-PD-1 cohort, there was no significant difference in survival time between different groups (**Figure 6J**).

### Correlation Between the Cancer-Associated Fibroblast-Based Signature and Somatic Variation

**Figure 7:**
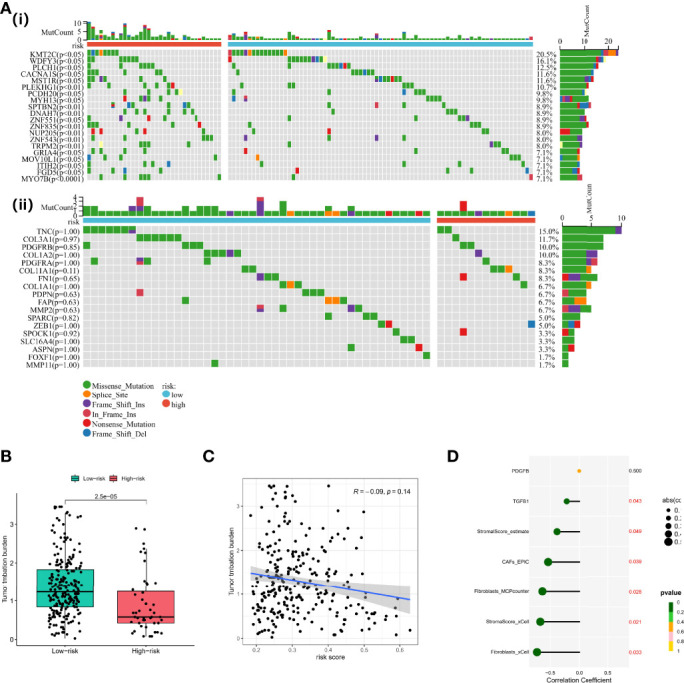
Functional analyses and construction of the CAF-based signature. **(A)** (i) Differentially mutated genes in different risk groups. (ii) CAF marker mutations in different risk groups. **(B)** TMB values in different risk groups. **(C)** Spearman analysis between CAF risk score and TMB values. **(D)** Correlation analysis between TMB values, stromal/CAF scores, and CAF-activating factors. CAFs, Cancer-Associated Fibroblasts; TMB, Tumor Mutational Burden.

Preclinical research has shown that patients with higher TMB are associated with enhanced immunotherapy response and lasting clinical benefits when treated with immune checkpoint blockade. Therefore, we investigated the discriminatory ability of the CAF-based signature in the somatic mutation data of TCGA-OV cohort. Firstly, we screened the most differentially mutated genes in different risk groups, including *KMT2C*, *WDFY3*, *CACNA1S*, etc. (**Figure 7A(i)**). We found no significant differences between the two groups in CAF marker mutations, but *TNC* (15.0%) and *COL3A*1 (11.7%) exhibited a higher frequency of mutations in the whole TCGA-OC cohort (**Figure 7A(ii)**). Subsequently, we observed that TMB values were higher in the low-risk group than those in the high-risk group (**Figure 7B**). However, Spearman analysis showed no statistically significant correlation between CAF risk score and TMB values (**Figure 7C**). However, TMB values were negatively correlated with stromal score and CAF-activating factors transforming growth factor beta (TGF-β), suggesting that higher TMB might have intense tumor-killing effects *via* modulating a fibroblast-weak TME (40) (**Figure 7D**).

### GSEA of the Cancer-Associated Fibroblast-Based Signature

**Figure 8:**
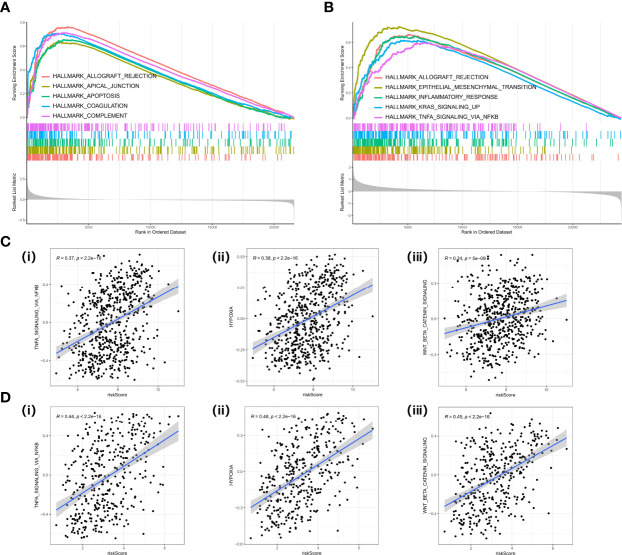
GSEA in cancer hallmark gene set. **(A)** GSEA plot in the GPL570 meta-cohort. **(B)** GSEA plot in TCGA-OV cohort. **(C)** Correlation analysis between risk score and TNF signaling (i), hypoxia (ii), EMT (iii) in the GPL570 meta-cohort. **(D)** Correlation analysis between risk score and TNF signaling (i), hypoxia (ii), and EMT (iii) in TCGA-OV cohort. GSEA, Gene Set Enrichment Analysis; TNF, Tumor Necrosis Factor; EMT, Epithelial to Mesenchymal Transition.

Gene Set Enrichment Analysis (GSEA) was performed in two datasets (GPL570 meta-cohort and TCGA-OV cohort) to explore the pathways involved in different risk groups. Allograft rejection, apical junction, and epithelial–mesenchymal transition were significantly enriched (**Figures 8A, B**). The ssGSEA score also showed that the CAF risk score was positively correlated with TNFA signaling *via* nuclear factor-kappaB (NF-kappaB), hypoxia, and Wnt beta catenin signaling pathway (**Figures 8C, D**).

### Sensitivity of Chemotherapy Between Different Risk Groups

**Figure 9:**
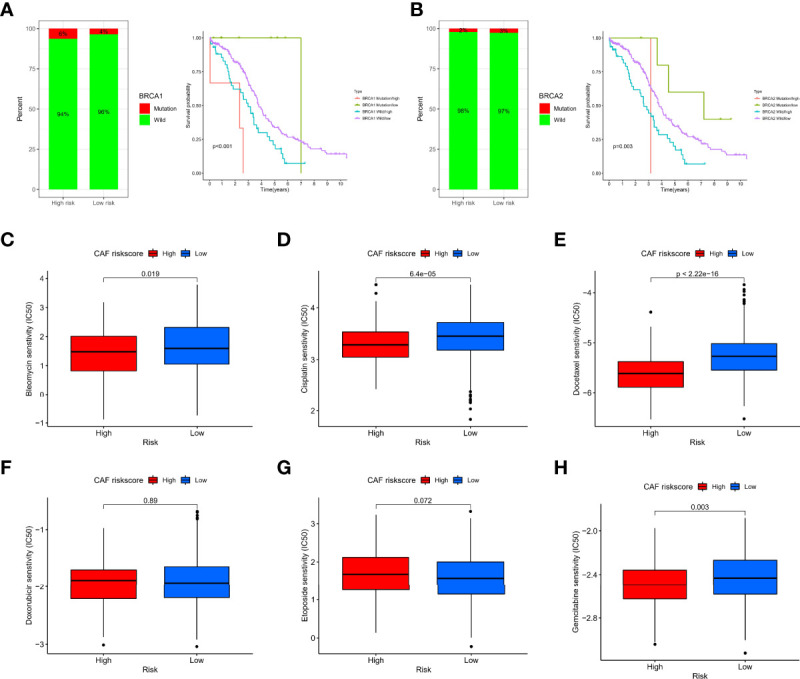
Sensitivity of chemotherapy between different risk groups. **(A)** Histogram of BRCA1 state distribution and Kaplan–Meier analysis of integrated groupings. **(B)** Histogram of BRCA2 state distribution and Kaplan–Meier analysis of integrated groupings. IC50 values between different risk groups, including bleomycin **(C)**, cisplatin **(D)**, docetaxel **(E)**, doxorubicin **(F)**, etoposide **(G)**, and gemcitabine **(H)**.

Maintenance therapy and chemotherapy after debulking surgery for OC patients are crucial, and the mutation of the Breast Cancer Susceptibility Genes (BRCA) is relevant to the efficacy of olaparib. Therefore, we explored the distribution of mutations in the BRCA under different risk groups. BRCA1 may be more distributed in the high-risk group, but there was no significant difference in BRCA2. Interestingly, the combined BRCA mutation status and risk score allowed for better survival prediction (**Figures 9A, B**). In addition, Wilcoxon analysis revealed significant differences in IC50 values between different risk groups. Among them, high-risk patients may be more sensitive to bleomycin (**Figure 9C**), cisplatin (**Figure 9D**), docetaxel (**Figure 9E**), and gemcitabine (**Figure 9H**). Still, the IC50 values of doxorubicin (**Figure 9F**) and etoposide (**Figure 9G**) were not significantly different between groups.

### Validation in Cell Lines, scRNA-Seq, and Immunohistochemistry

**Figure 10:**
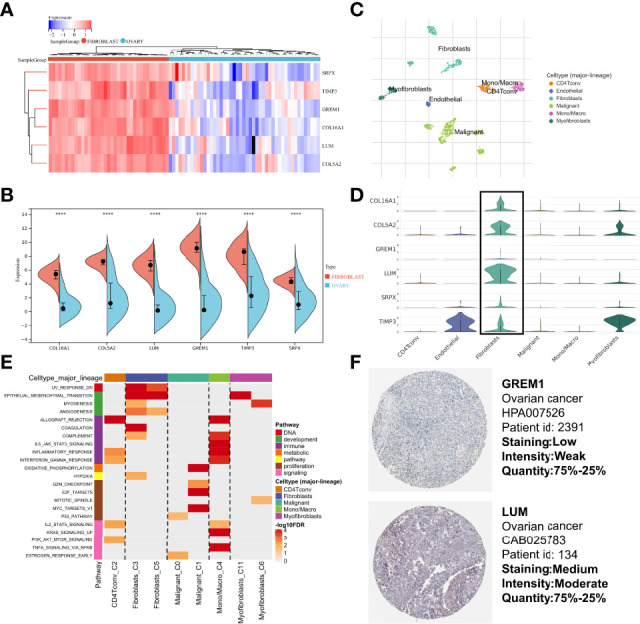
Multidimensional expression validation. **(A)** Heatmap of gene expression in different cell lines based on “limma” package. **(B)** Wilcoxon test of gene expression in different cell lines. **(C)** Major cell type in single-cell seq. **(D)** Differential distribution of gene expression at the single-cell level. **(E)** GSEA of upregulated genes in different cell types. **(F)** IHC images of OC tissues from the HPA database. GSEA, Gene Set Enrichment Analysis; OC, Ovarian cancer; IHC, Immunohistochemistry; HPA, Human Protein Atlas.

To validate that the CAF-related genes involved in the signature were the primary origins in CAFs, we performed a multidimensional validation, including cell lines, single-cell sequencing, and immunohistochemistry. We collected cell line RNA-seq data from 47 fibroblast origins and 37 OC origins. We found that all six genes (COL16A1, COL5A2, GREM1, LUM, SRPX, and TIMP3) were overexpressed in fibroblasts by the “limma” package (**Figure 10A**) and Wilcoxon test (**Figure 10B**). Meanwhile, we annotated the scRNA-seq into 6 clusters: fibroblasts, myofibroblasts, endothelial, malignant, Mono/Macro, and CD4Tconv (**Figure 10C**). The differential analysis results showed that most CAF-related genes were highly expressed in fibroblasts or myofibroblasts, while lower expression was observed in malignant (**Figure 10D**). Moreover, the single-cell GSEA was consistent with the bulk-RNA GSEA, showing significant enrichment of upregulated genes of fibroblasts in the EMT pathway (**Figure 10E**). We analyzed IHC images from the HPA database, and the section showed that GREM1 and LUM proteins were deeply stained in the stroma (**Figure 10F**). Unfortunately, the other four genes did not have corresponding IHC images in the HPA database. These verifications implied that these six genes might be CAF-specific markers.

## Discussion

The CAF is regarded as an essential factor in promoting tumor progression by interacting with cancer cells in the TME (1). Meanwhile, for a specific mesenchymal subtype of OC, it is characterized by frequent generation of desmoplastic stroma (41). The generation of desmoplastic stroma is associated with a lower OS and resistance to platinum (42). Consistently, we observed that higher CAF and stromal scores were associated with poorer OS in OC patients and represented a poorer immunotherapy response. This is the first study with a large sample and using WGCNA as a starting point for exploring markers associated with CAFs. A 6-gene prognostic (COL16A1, COL5A2, GREM1, LUM, SRPX, and TIMP3) signature was constructed and validated using Cox and LASSO regression algorithms. With the cutoff value as a threshold, we observed that patients with a high CAF risk score were more sensitive to numerous chemotherapeutic agents. Furthermore, we revealed that lower risk scores were associated with improved immunotherapy outcomes and higher TMB value. Based on our results, we propose an alternative mechanism by which higher TMB may also enhance tumor killing by modulating the microenvironment of stromal fibroblasts, similar to previous findings. It reported that cancer cells with high levels of somatic mutation are more easily recognized by the immune system (43). However, we need more *in vitro* and *in vivo* experiments to elucidate the above crosstalk in the future.

Compared to the traditional differential gene expression (DEG) approach for screening hub CAF markers (44), we used different bioinformatics algorithms to assess the abundance of CAFs and biomarkers in each OC sample to ensure the robustness (EPIC and MCP-counter for WGCNA network construction; xCell and ESTIMATE for correlation validation). Similarly, to ensure the robustness of the prognostic signature, different cohorts were used for construction and validation (GPL570 meta-cohort for construction; TCGA-OV cohort, GPL96 meta-cohort, GPL7759 cohort, GPL2986 cohort, GPL14951 cohort, IMvigor210 cohort, and GSE78220 cohort for validation). With the above approach, we confirmed that our model closely correlated with CAF infiltration and CAF markers from references. Meanwhile, to differentiate identified genes from tumor cells to highlight gene heterogeneity in CAFs, we confirmed significantly higher expression in fibroblast cell lines, higher staining of proteins in the stroma, and higher mRNA expression of the CAFs at the single-cell level.

For the six genes involved in the risk signature, the relevant references have reported on the role in tumor cells and TME. COL16A1 was indicated in the study of Pan and Ma (45) to be involved in a risk model and could be considered a prognostic marker in OC patients. Renner et al. (46) sought to determine the ECM composition of benign fallopian tubes and the changes associated with tubal intraepithelial carcinomas and identified seven proteins that had not been identified in previous studies (COL2A1, COL4A5, COL16A1, elastin, LAMA5, annexin A2, and PAI1). Interestingly, they suggested that the seven proteins mentioned above accompany tubal intraepithelial carcinoma formation and cause ECM changes. Head and neck squamous cell carcinoma (HNSCC) cell lines were cocultured with their patient-matched CAFs in 2D and 3D *in vitro* models, and GREM1 was upregulated (47). In addition, related studies have also found that GREM1 binds to miR-205-5p (48) or miR-206 (49) to regulate metastasis of cervical cancer and non-small cell carcinoma. As part of the ECM, collagen family proteins, together with elastins, fibronectins, and laminins, play a key role in tissue organization, tissue resistance, and its primary shape. The collagen family, including COL5A2, is overexpressed in various types of epithelial cancers and is associated with poorer OS. Furthermore, inhibition of gene expression decreases cell proliferation and invasion (50, 51). SRPX, also known as SRPX1 (52), ETX1 (53), and DRS (54), is a suppressor that has been found to be downregulated in a range of human tumor cells and tissues. Unlike other soluble members of the Tissue Inhibition of Matrix Metalloproteinase (TIMP) family, TIMP3 is tightly sequestered in the ECM. TIMP-3 is also the only TIMP capable of inhibiting tumor necrosis factor alpha (TNF-α), ADAMTS4, and ADAMTS5, as well as syndecan sheddase (55). Nevertheless, functional validation about the six genes involved in the risk signature in the CAFs of OC is not much, which requires us to conduct further experiments on the six CAF markers in the future.

In conclusion, the CAF risk score can be used in clinical practice to comprehensively evaluate the corresponding cellular infiltration of CAFs in patients to further define the immunophenotype. We have also demonstrated that risk score can be used to assess the clinicopathological characteristics of patients. Similarly, risk score also can be used as a biomarker to predict survival and the efficacy of adjuvant chemotherapy and the response to anti-PD-1/PD-L1 immunotherapy. More importantly, this study may help to leverage the future development of new drug combination strategies or new immunotherapeutic agents. Our findings provide new ideas to facilitate future individualized cancer immunotherapy.

## Data Availability Statement

The original contributions presented in the study are included in the article/**Supplementary Material**. Further inquiries can be directed to the corresponding author.

## Ethics Statement

The studies involving human participants were reviewed and approved by the Ethics Committees and Institutional Review Boards of Zhongda Hospital Southeast University (ZDSYLL187-P04). The patients/participants provided their written informed consent to participate in this study. Written informed consent was obtained from the individual(s) for the publication of any potentially identifiable images or data included in this article.

## Author Contributions

SF, YX, and ZD conceived and designed the study. SY was responsible for materials. SF drafted the article. SY, HY, and KZ revised the article critically. All authors had final approval of the submitted versions.

## Funding

This study was supported by National Natural Science Foundation of China (No. 82072078), Jiangsu Province Key Research and Development Project (SBE2020741118), and Postgraduate Research & Practice Innovation Program of Jiangsu Province (SJCX22_0070).

## Conflict of Interest

The authors declare that the research was conducted in the absence of any commercial or financial relationships that could be construed as a potential conflict of interest.

## Publisher’s Note

All claims expressed in this article are solely those of the authors and do not necessarily represent those of their affiliated organizations, or those of the publisher, the editors and the reviewers. Any product that may be evaluated in this article, or claim that may be made by its manufacturer, is not guaranteed or endorsed by the publisher.

## Supplementary Material

Supplementary Figure 1 Common hub genes in different dataset.

Supplementary Figure 2 Normalization process based on the GPL97 platform dataset. **(A)** UMAP plot of the four datasets before normalization. **(B)** UMAP plot of the four datasets after normalization. **(C)** Expression density plot of the six datasets before normalization. **(D)** Expression density plot of the four datasets after normalization. **(E)** Expression distribution plots for the four datasets after normalization.

## References

[1] De P Aske J Dey N. Cancer-Associated Fibroblast Functions as a Road-Block in Cancer Therapy. Cancers (Basel) (2021) 13(20):5246. doi: 10.3390/cancers13205246 34680395 PMC8534063

[2] Ghoneum A Afify H Salih Z Kelly M Said N. Role of Tumor Microenvironment in the Pathobiology of Ovarian Cancer: Insights and Therapeutic Opportunities. Cancer Med (2018) 7(10):5047–56. doi: 10.1002/cam4.1741 PMC6198242 30133163

[3] Johnson RL Cummings M Thangavelu A Theophilou G de Jong D Orsi NM. Barriers to Immunotherapy in Ovarian Cancer: Metabolic, Genomic, and Immune Perturbations in the Tumour Microenvironment. Cancers (Basel) (2021) 13(24):6231. doi: 10.3390/cancers13246231 34944851 PMC8699358

[4] Lakins MA Ghorani E Munir H Martins CP Shields JD. Cancer-Associated Fibroblasts Induce Antigen-Specific Deletion of CD8 + T Cells to Protect Tumour Cells. Nat Commun (2018) 9(1):948. doi: 10.1038/s41467-018-03347-0 29507342 PMC5838096

[5] Gamradt P de la Fouchardière C Hennino A. Stromal Protein-Mediated Immune Regulation in Digestive Cancers. Cancers (Basel) (2021) 13(1):146. doi: 10.3390/cancers13010146 PMC7795083 33466303

[6] Shin K Lim A Zhao C Sahoo D Pan Y Spiekerkoetter E. Hedgehog Signaling Restrains Bladder Cancer Progression by Eliciting Stromal Production of Urothelial Differentiation Factors. Cancer Cell (2014) 26(4):521–33. doi: 10.1016/j.ccell.2014.09.001 PMC4326077 25314078

[7] Yoshida GJ. Regulation of Heterogeneous Cancer-Associated Fibroblasts: The Molecular Pathology of Activated Signaling Pathways. J Exp Clin Cancer Res (2020) 39(1):112. doi: 10.1186/s13046-020-01611-0 32546182 PMC7296768

[8] Huang TX Tan XY Huang HS Li YT Liu BL Liu KS. Targeting Cancer-Associated Fibroblast-Secreted WNT2 Restores Dendritic Cell-Mediated Antitumour Immunity. Gut (2022) 71(2):333–44. doi: 10.1136/gutjnl-2020-322924 PMC8762012 33692094

[9] Zhao X Ding L Lu Z Huang X Jing Y Yang Y. Diminished CD68+ Cancer-Associated Fibroblast Subset Induces Regulatory T-Cell (Treg) Infiltration and Predicts Poor Prognosis of Oral Squamous Cell Carcinoma Patients. Am J Pathol (2020) 190(4):886–99. doi: 10.1016/j.ajpath.2019.12.007 32035062

[10] Duperret EK Trautz A Ammons D Perales-Puchalt A Wise MC Yan J. Alteration of the Tumor Stroma Using a Consensus DNA Vaccine Targeting Fibroblast Activation Protein (FAP) Synergizes With Antitumor Vaccine Therapy in Mice. Clin Cancer Res (2018) 24(5):1190–201. doi: 10.1158/1078-0432.CCR-17-2033 PMC5844837 29269377

[11] Raskov H Orhan A Gaggar S Gögenur I. Cancer-Associated Fibroblasts and Tumor-Associated Macrophages in Cancer and Cancer Immunotherapy. Front Oncol (2021) 11:668731. doi: 10.3389/fonc.2021.668731 34094963 PMC8172975

[12] Langfelder P Horvath S. WGCNA: An R Package for Weighted Correlation Network Analysis. BMC Bioinf (2008) 9:559. doi: 10.1186/1471-2105-9-559 PMC2631488 19114008

[13] Zheng H Liu H Li H Dou W Wang X. Weighted Gene Co-Expression Network Analysis Identifies a Cancer-Associated Fibroblast Signature for Predicting Prognosis and Therapeutic Responses in Gastric Cancer. Front Mol Biosci (2021) 8:744677. doi: 10.3389/fmolb.2021.744677 34692770 PMC8531434

[14] Liu B Zhan Y Chen X Hu X Wu B Pan S. Weighted Gene Co-Expression Network Analysis can Sort Cancer-Associated Fibroblast-Specific Markers Promoting Bladder Cancer Progression. J Cell Physiol (2021) 236(2):1321–31. doi: 10.1002/jcp.29939 32657439

[15] Liu B Chen X Zhan Y Wu B Pan S. Identification of a Gene Signature for Renal Cell Carcinoma-Associated Fibroblasts Mediating Cancer Progression and Affecting Prognosis. Front Cell Dev Biol (2021) 8:604627. doi: 10.3389/fcell.2020.604627 33634098 PMC7901886

[16] Goldman MJ Craft B Hastie M Repečka K McDade F Kamath A. Visualizing and Interpreting Cancer Genomics Data *via* the Xena Platform. Nat Biotechnol (2020) 38(6):675–8. doi: 10.1038/s41587-020-0546-8 PMC7386072 32444850

[17] Cibulskis K Lawrence MS Carter SL Sivachenko A Jaffe D Sougnez C. Sensitive Detection of Somatic Point Mutations in Impure and Heterogeneous Cancer Samples. Nat Biotechnol (2013) 31(3):213–9. doi: 10.1038/nbt.2514 PMC3833702 23396013

[18] Wagner GP Kin K Lynch VJ. Measurement of mRNA Abundance Using RNA-Seq Data: RPKM Measure is Inconsistent Among Samples. Theory Biosci (2012) 131(4):281–5. doi: 10.1007/s12064-012-0162-3 22872506

[19] Barrett T Edgar R. Gene Expression Omnibus: Microarray Data Storage, Submission, Retrieval, and Analysis. Methods Enzymol (2006) 411:352–69. doi: 10.1016/S0076-6879(06)11019-8 PMC1619900 16939800

[20] Ghandi M Huang FW Jané-Valbuena J Kryukov GV Lo CC McDonald ER 3rd. Next-Generation Characterization of the Cancer Cell Line Encyclopedia. Nature (2019) 569(7757):503–8. doi: 10.1038/s41586-019-1186-3 PMC6697103 31068700

[21] Uhlén M Fagerberg L Hallström BM Lindskog C Oksvold P Mardinoglu A. Proteomics. Tissue-Based Map of the Human Proteome. Science (2015) 347(6220):1260419. doi: 10.1126/science.1260419 25613900

[22] Leek JT Johnson WE Parker HS Jaffe AE Storey JD. The Sva Package for Removing Batch Effects and Other Unwanted Variation in High-Throughput Experiments. Bioinformatics (2012) 28(6):882–3. doi: 10.1093/bioinformatics/bts034 PMC3307112 22257669

[23] Han C Liu T Yin R. Biomarkers for Cancer-Associated Fibroblasts. biomark Res (2020) 8(1):64. doi: 10.1186/s40364-020-00245-w 33292666 PMC7661188

[24] Racle J de Jonge K Baumgaertner P Speiser DE Gfeller D. Simultaneous Enumeration of Cancer and Immune Cell Types From Bulk Tumor Gene Expression Data. Elife (2017) 6:e26476. doi: 10.7554/eLife.26476 29130882 PMC5718706

[25] Aran D Hu Z Butte AJ. Xcell: Digitally Portraying the Tissue Cellular Heterogeneity Landscape. Genome Biol (2017) 18(1):220. doi: 10.1186/s13059-017-1349-1 29141660 PMC5688663

[26] Becht E Giraldo NA Lacroix L Buttard B Elarouci N Petitprez F. Estimating the Population Abundance of Tissue-Infiltrating Immune and Stromal Cell Populations Using Gene Expression. Genome Biol (2016) 17(1):218. doi: 10.1186/s13059-016-1070-5 27765066 PMC5073889

[27] Yoshihara K Shahmoradgoli M Martínez E Vegesna R Kim H Torres-Garcia W. Inferring Tumour Purity and Stromal and Immune Cell Admixture From Expression Data. Nat Commun (2013) 4:2612. doi: 10.1038/ncomms3612 24113773 PMC3826632

[28] Zeng D Ye Z Shen R Yu G Wu J Xiong Y. IOBR: Multi-Omics Immuno-Oncology Biological Research to Decode Tumor Microenvironment and Signatures. Front Immunol (2021) 12:687975. doi: 10.3389/fimmu.2021.687975 34276676 PMC8283787

[29] Shen X Yang Z Feng S Li Y. Identification of Uterine Leiomyosarcoma-Associated Hub Genes and Immune Cell Infiltration Pattern Using Weighted Co-Expression Network Analysis and CIBERSORT Algorithm. World J Surg Oncol (2021) 19(1):223. doi: 10.1186/s12957-021-02333-z 34321013 PMC8320213

[30] Liberzon A Birger C Thorvaldsdóttir H Ghandi M Mesirov JP Tamayo P. The Molecular Signatures Database (MSigDB) Hallmark Gene Set Collection. Cell Syst (2015) 1(6):417–25. doi: 10.1016/j.cels.2015.12.004 PMC4707969 26771021

[31] Hänzelmann S Castelo R Guinney J. GSVA: Gene Set Variation Analysis for Microarray and RNA-Seq Data. BMC Bioinf (2013) 14:7. doi: 10.1186/1471-2105-14-7 PMC3618321 23323831

[32] Yu G Wang LG Han Y He QY. Clusterprofiler: An R Package for Comparing Biological Themes Among Gene Clusters. OMICS (2012) 16(5):284–7. doi: 10.1089/omi.2011.0118 PMC3339379 22455463

[33] Simon N Friedman J Hastie T Tibshirani R. Regularization Paths for Cox's Proportional Hazards Model *via* Coordinate Descent. J Stat Software (2011) 39(5):1–13. doi: 10.18637/jss.v039.i05 PMC4824408 27065756

[34] Feng S Yin H Zhang K Shan M Ji X Luo S. Integrated Clinical Characteristics and Omics Analysis Identifies a Ferroptosis and Iron-Metabolism-Related lncRNA Signature for Predicting Prognosis and Therapeutic Responses in Ovarian Cancer. J Ovarian Res (2022) 15(1):10. doi: 10.1186/s13048-022-00944-y 35057848 PMC8772079

[35] Zhu Y Feng S Song Z Wang Z Chen G. Identification of Immunological Characteristics and Immune Subtypes Based on Single-Sample Gene Set Enrichment Analysis Algorithm in Lower-Grade Glioma. Front Genet (2022) 13:894865. doi: 10.3389/fgene.2022.894865 35646050 PMC9136245

[36] Geeleher P Cox N Huang RS. Prrophetic: An R Package for Prediction of Clinical Chemotherapeutic Response From Tumor Gene Expression Levels. PloS One (2014) 9(9):e107468. doi: 10.1371/journal.pone.0107468 25229481 PMC4167990

[37] Sun D Wang J Han Y Dong X Ge J Zheng R. TISCH: A Comprehensive Web Resource Enabling Interactive Single-Cell Transcriptome Visualization of Tumor Microenvironment. Nucleic Acids Res (2021) 49(D1):D1420–30. doi: 10.1093/nar/gkaa1020 PMC7778907 33179754

[38] Zhao S Liang T Zhang C Shi D Jiang W Su C. IL-27 Rα+ Cells Promoted Allorejection *via* Enhancing STAT1/3/5 Phosphorylation. J Cell Mol Med (2020) 24(18):10756–67. doi: 10.1111/jcmm.15700 PMC7521268 32761753

[39] Pernot S Evrard S Khatib AM. The Give-And-Take Interaction Between the Tumor Microenvironment and Immune Cells Regulating Tumor Progression and Repression. Front Immunol (2022) 13:850856. doi: 10.3389/fimmu.2022.850856 35493456 PMC9043524

[40] Pietras K Pahler J Bergers G Hanahan D. Functions of Paracrine PDGF Signaling in the Proangiogenic Tumor Stroma Revealed by Pharmacological Targeting. PloS Med (2008) 5(1):e19. doi: 10.1371/journal.pmed.0050019 18232728 PMC2214790

[41] Tothill RW Tinker AV George J Brown R Fox SB Lade S. Novel Molecular Subtypes of Serous and Endometrioid Ovarian Cancer Linked to Clinical Outcome. Clin Cancer Res (2008) 14(16):5198–208. doi: 10.1158/1078-0432.CCR-08-0196 18698038

[42] Verhaak RG Tamayo P Yang JY Hubbard D Zhang H Creighton CJ. Prognostically Relevant Gene Signatures of High-Grade Serous Ovarian Carcinoma. J Clin Invest (2013) 123(1):517–25. doi: 10.1172/JCI65833 PMC3533304 23257362

[43] Miao D Margolis CA Vokes NI Liu D Taylor-Weiner A Wankowicz SM. Genomic Correlates of Response to Immune Checkpoint Blockade in Microsatellite-Stable Solid Tumors. Nat Genet (2018) 50(9):1271–81. doi: 10.1038/s41588-018-0200-2 PMC6119118 30150660

[44] Kim MJ Jung D Park JY Lee SM An HJ. GLIS1 in Cancer-Associated Fibroblasts Regulates the Migration and Invasion of Ovarian Cancer Cells. Int J Mol Sci (2022) 23(4):2218. doi: 10.3390/ijms23042218 35216340 PMC8874490

[45] Pan X Ma X. A Novel Six-Gene Signature for Prognosis Prediction in Ovarian Cancer. Front Genet (2020) 11:1006. doi: 10.3389/fgene.2020.01006 33193589 PMC7593580

[46] Renner C Gomez C Visetsouk MR Taha I Khan A McGregor SM. Multi-Modal Profiling of the Extracellular Matrix of Human Fallopian Tubes and Serous Tubal Intraepithelial Carcinomas. J Histochem Cytochem (2022) 70(2):151–68. doi: 10.1369/00221554211061359 PMC8777377 34866441

[47] Wiechec E Magan M Matic N Ansell-Schultz A Kankainen M Monni O. Cancer-Associated Fibroblasts Modulate Transcriptional Signatures Involved in Proliferation, Differentiation and Metastasis in Head and Neck Squamous Cell Carcinoma. Cancers (Basel) (2021) 13(13):3361. doi: 10.3390/cancers13133361 34283070 PMC8269044

[48] Kan J Fu B Zhou R Zhou D Huang Y Zhao H. He-Chan Pian Inhibits the Metastasis of non-Small Cell Lung Cancer *via* the miR-205-5p-Mediated Regulation of the GREM1/Rap1 Signaling Pathway. Phytomedicine (2022) 94:153821. doi: 10.1016/j.phymed.2021.153821 34752967

[49] Sun Q Qi X Zhang W Li X. Knockdown of circRNA_0007534 Suppresses the Tumorigenesis of Cervical Cancer *via* miR-206/GREM1 Axis. Cancer Cell Int (2021) 21(1):54. doi: 10.1186/s12935-021-01749-7 33446214 PMC7809877

[50] Ding YL Sun SF Zhao GL. COL5A2 as a Potential Clinical Biomarker for Gastric Cancer and Renal Metastasis. Med (Baltimore) (2021) 100(7):e24561. doi: 10.1097/MD.0000000000024561 PMC7899835 33607786

[51] Ren X Chen X Fang K Zhang X Wei X Zhang T. COL5A2 Promotes Proliferation and Invasion in Prostate Cancer and Is One of Seven Gleason-Related Genes That Predict Recurrence-Free Survival. Front Oncol (2021) 11:583083. doi: 10.3389/fonc.2021.583083 33816226 PMC8012814

[52] Inoue Y Ueda M Tasaki M. Sushi Repeat-Containing Protein 1: A Novel Disease-Associated Molecule in Cerebral Amyloid Angiopathy. Acta Neuropathol (2017) 134(4):605–17. doi: 10.1007/s00401-017-1720-z 28478503

[53] Iragavarapu S Algeciras ME Lee RK Bhattacharya SK. ETX1 is Over-Expressed in the Glaucomatous Trabecular Meshwork. Mol Vis (2009) 15:2061–7. PMC2765241 19862339

[54] Tambe Y Hasebe M Kim CJ Yamamoto A Inoue H. The Drs Tumor Suppressor Regulates Glucose Metabolism *via* Lactate Dehydrogenase-B. Mol Carcinog (2016) 55(1):52–63. doi: 10.1002/mc.22258 25620379

[55] Qi JH Bell B Singh R Batoki J Wolk A Cutler A. Sorsby Fundus Dystrophy Mutation in Tissue Inhibitor of Metalloproteinase 3 (TIMP3) Promotes Choroidal Neovascularization *via* a Fibroblast Growth Factor-Dependent Mechanism. Sci Rep (2019) 9(1):17429. doi: 10.1038/s41598-019-53433-6 31757977 PMC6874529

