# Supplementary figures and images for: Integrative Analysis From Multicenter Studies Identifies a WGCNA-Derived Cancer-Associated Fibroblast Signature for Ovarian Cancer
Source: Front Immunol. 2022 Jul 8;13:951582. doi: 10.3389/fimmu.2022.951582 (PMC9304893; doi:10.3389/fimmu.2022.951582)

**A****GPL96**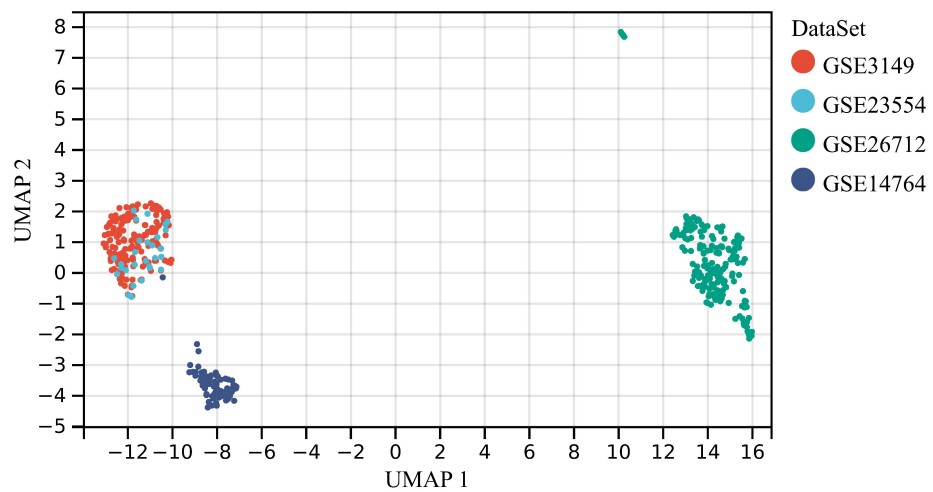**B**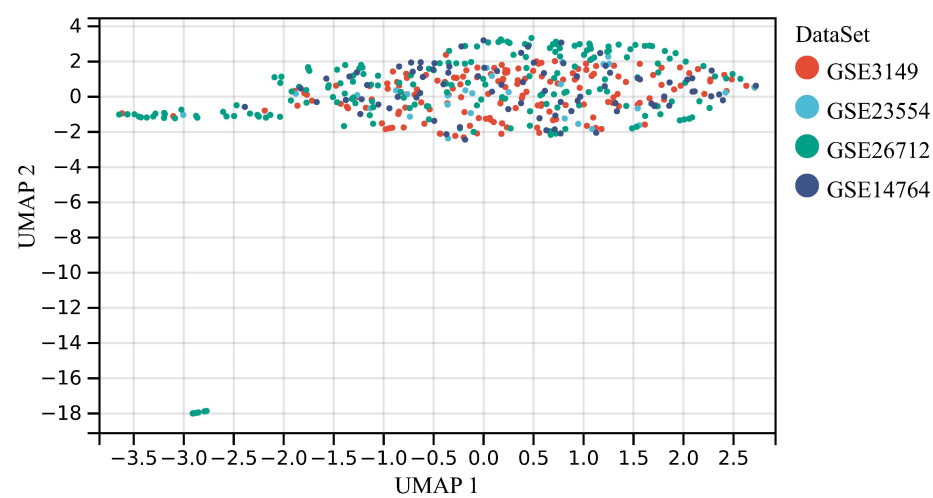**C**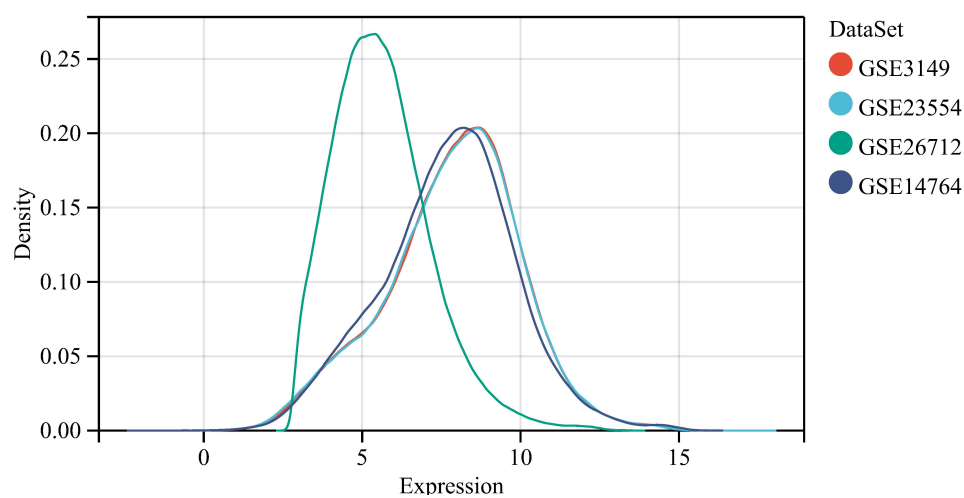**D**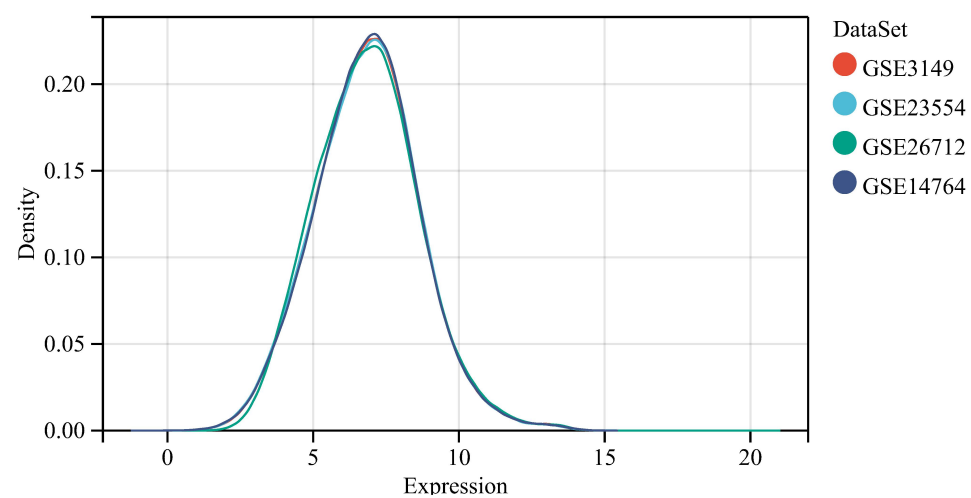**E**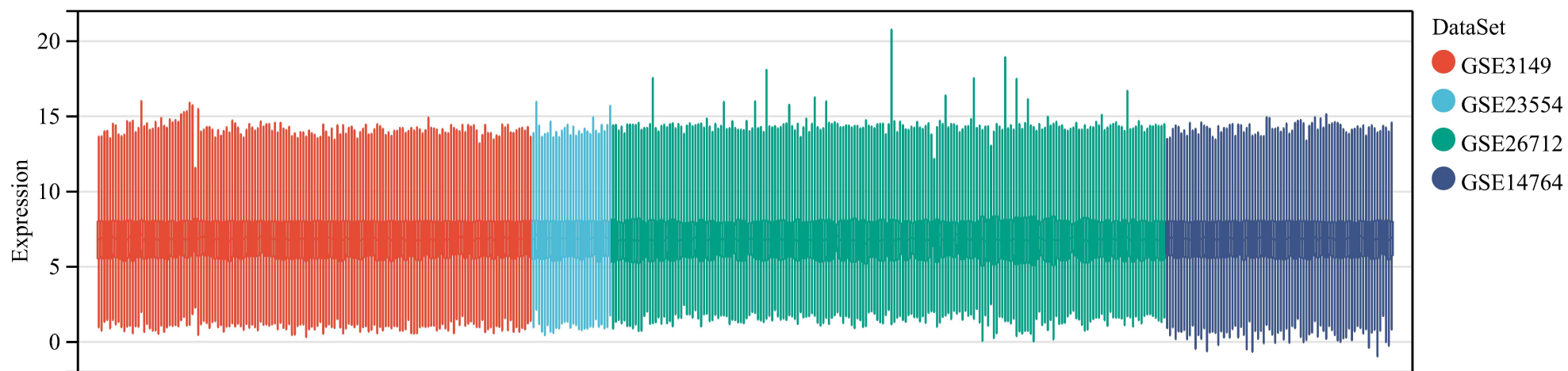

Supplement: Supplementary Figure 1 — Common hub genes in different dataset. [file DataSheet_1.pdf]

Intersection Size

60

40

20

0

2

3

4

2

6

4

3

71

GPL14951

GPL7759

GPL2986

GPL96

TCGA

GPL570

75

50

25

0

Set Size

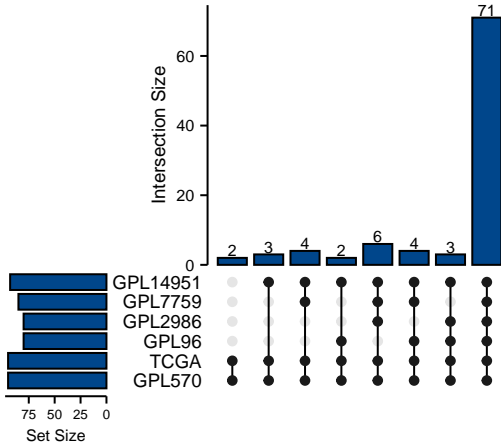

Supplement: Supplementary Figure 2 — Normalization process based on the GPL97 platform dataset. (A) UMAP plot of the four datasets before normalization. (B) UMAP plot of the four datasets after normalization. (C) Expression density plot of the six datasets before normalization. (D) Expression density plot of the four datasets after normalization. (E) Expression distribution plots for the four datasets after normalization. [file DataSheet_2.pdf]
